# Architectural Ultrasound Pennation Angle Measurement of Lumbar Multifidus Muscles: A Reliability Study

**DOI:** 10.3390/jcm11175174

**Published:** 2022-09-01

**Authors:** Cristina Monsalve-Vicente, Daniel Muñoz-Zamarro, Nicolás Cuenca-Zaldívar, Samuel Fernández-Carnero, Francisco Selva-Sarzo, Susana Nunez-Nagy, Fermin Naranjo-Cinto, Tomás Gallego-Izquierdo

**Affiliations:** 1 Universidad de Alcalá, Facultad de Medicina y Ciencias de la Salud, Departamento de Enfermería y Fisioterapia, Grupo de Investigación en Fisioterapia y Dolor, 28801 Alcalá de Henares, Spain; 2 Research Group in Nursing and Health Care, Puerta de Hierro Health Research Institute–Segovia de Arana (IDIPHISA), 28222 Madrid, Spain; 3 Department of Physiotherapy, University of Valencia, 46010 Valencia, Spain

**Keywords:** ultrasound, back muscles, pennation angle, reliability

## Abstract

The pennation angle has been shown to be a relevant parameter of muscle architecture. This parameter has not previously been measured in the lumbar multifidus musculature, and it is for this reason that it has been considered of great interest to establish an assessment protocol to generate new lines of research in the future. Objective: The objective of this study was to establish a protocol for measuring the pennation angle of the multifidus muscles, with a study of intra-rater and interrater reliability values. Design: This was a reliability study following the recommendations of the Guidelines for Reporting Reliability and Agreement Studies (GRRAS). Setting: The study was carried out at University of Alcalá, Department of Physiotherapy. Subjects: Twenty-seven subjects aged between 18 and 55 years were recruited for this study. Methods: Different ultrasound images of the lumbar multifidus musculature were captured. Subsequently, with the help of ImageJ software, the pennation angle of this musculature was measured. Finally, a complex statistical analysis determined the intra- and interrater reliability. Results: The intra-rater reliability of the pennation angle measurement protocol was excellent for observer 1 in the measurement of the left-sided superficial multifidus 0.851 (0.74, 0.923), and for observer 2 in the measurement of the right-sided superficial 0.711 (0.535, 0.843) and deep multifidus 0.886 (0.798, 0.942). Interrater reliability was moderate to poor, and correlation analysis results were high for thickness vs. pennation angle. Conclusions: The designed protocol for ultrasound measurement of the pennation angle of the lumbar multifidus musculature has excellent intra-rater reliability values, supporting the main conclusions and interpretations. Normative ranges of pennation angles are reported. High correlation between variables is described.

## 1. Introduction

The function of a muscle is largely determined by its muscle architecture [1], and the study of this ultrasound muscle parameter has been published in relation to different human situations, highly related to its function [2], opening new opportunities to study the impact of pathological situations in clinical settings [3].

Indeed, muscle architecture has been used to evaluate the impact of interventions because it has been demonstrated to be modified during the treatment (e.g., therapy based on exercise) [4].

Significant relationships exist among architectural parameters, and these parameters are predictive of a muscle’s force-generating capacity [2]. It is noteworthy that numerous studies have shown changes in muscle architecture associated with painful pathological processes [5,6,7,8].

One of the main techniques used to assess muscle architecture in vivo is conventional B-mode ultrasound [9].

The main muscle architecture parameters that can be measured by ultrasound are the length of muscle fascicles; the angle of pennation (PA) [10], which is defined as the angle at which the muscle fiber is arranged with respect to the force-generating axis of the muscle [1]; and muscle thickness (MT) [11]. 

The limitations of this technique are that measurements depend on the operator, the skill of the sonographer, and the orientation of the probe. A change in the orientation and rotation of the ultrasound probe can result in a 12% difference in the reported pennation angle [12]. Studies have shown that ultrasound has moderate-to-excellent reliability, with intraclass correlation values ranging from 0.72 to 0.98 for the measurement of lumbar paravertebral muscle cross-sectional area (CSA), fascicle length, and muscle thickness [13].

It has been observed that people with back pain have altered function of the lumbar extensors and changes in muscle morphology [14], while normal subjects exhibit a 23% change in muscle thickness during contraction [15].

In particular, the lumbar multifidus (LM) muscles play an important role in chronic non-specific low back pain [16]. Structural alterations in this musculature have been found in subjects with low back pain [5], as well as increased activity [17]. A review by Fortin and Macedo concludes that the multifidus musculature is significantly smaller in patients with chronic low back pain [6].

Measurement of the pennation angle of the lumbar multifidus muscles has never been performed before. Additionally, the correlation between this variable and baseline population characteristics (as studied previously in other muscles [18]) has not previously been measured with the aim of establishing the normal values and comparing them with pathological cases.

Based on this premise, the purpose of this study was to establish a protocol for measuring the pennation angle of the superficial multifidus and deep multifidus muscles in a resting situation, and to assess the intra-rater and interrater reliability. A further goal of this study was to test the existence of a correlation between pennation angle, muscle thickness, and the different basal variables of both superficial and deep multifidus musculature.

## 2. Materials and Methods

An intra-rater and interrater reliability study was carried out with healthy subjects. The study was conducted at the Faculty of Nursing and Physiotherapy of the University of Alcalá (Madrid). The study followed the recommendations of the Guidelines for Reporting Reliability and Agreement Studies (GRRAS) [19] and the Declaration of Helsinki [20]. It was approved by the Ethics Committee of the University of Alcalá (No. CEIM/HU/2020/52). All participants were informed, verbally and in writing, of the procedure that was going to be carried out, that they would have to sign an informed consent agreement to participate in the study, and that the rights of human subjects were protected.

The study sample consisted of subjects who met the inclusion criterion and who did not meet any exclusion criteria. For inclusion, participants had to be aged between 18 and 55 years. The exclusion criteria were: having suffered localized low back pain or pain referring to other regions (e.g., pelvic or hip pain), in the previous 3 months; diagnosis of any structural alteration of the spine such as hernia, spondylolisthesis, stenosis of the spinal canal, etc.; presence of neurological disease; previous spinal surgery; professional sports practice; presence of comorbidities that could produce muscular affectation such as diabetes or cardio-respiratory diseases; and the presence of dyspnea/post-COVID-19 deconditioning.

To calculate the sample size, the formula proposed by Zou (2012) [21] was used, taking the interobserver ICC2 between the averages of the pennation angle in the superficial and deep multifidus of the first 10 subjects recruited in the study. The sample size N is defined by the formula:

$$N=1+\frac{2\cdot {\left(z_{\alpha }+z_{\beta }\right)}^{2}\cdot k}{\ln{\left[F\left(p\right)/F\left(p_{0}\right)\right]}^{2}\cdot \left(k-1\right)}$$

where z_α_ is the upper quantile of the standard normal distribution, z_β_ is the upper β quantile of the standard normal distribution, k is the number of observations used, F(p) is the F-distribution of the calculated ICC2, and F(p_0_) the F-distribution of the expected value for the ICC2 null hypothesis.

With the first 10 subjects recruited, a final sample of 27 subjects was estimated, accepting a risk of 0.05 (type I error) and a power of 80% (20% type II error).

### Procedure

**Figure 1:**
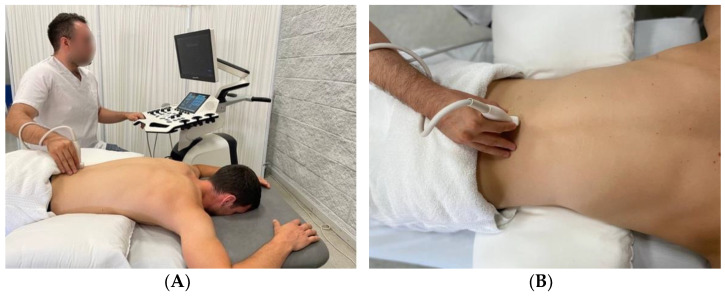
Ultrasound imaging sampling setting. (**A**) Patient lying prone, sonographer close to the patient and the ultrasound machine; (**B**) detailed image with the probe parasagittal over the lamina.

For assessment by ultrasound imaging, the subject was placed in a prone- lying position, with the lumbar curve flattened to less than 10°, using a pillow placed under the abdomen, and the arms in 90° shoulder abduction and elbow flexion, so that the elbows hung off the stretcher [13,17,22,23] and the face was supported in the bed hole (Figure 1).

The ultrasound measurements were made by a VINNO E35 ultrasound device with a linear 5–13 MHz probe and a 48 mm footprint.

Regarding the location of the probe, based on the anatomy of the superficial and deep multifidus musculature [24], it was placed on a long axis parallel to the vertebral column on the spinous process of any of the lumbar vertebrae. From that position, the probe was moved laterally until the lamina of that vertebra was located on the screen. It was then moved caudally to view the sacral bone, and then moved cranially until the laminae corresponding to the L5 and L4 vertebrae appeared on the image. Following this process, in which the sacral bone was taken as a reference, it was possible to ensure that all images obtained corresponded to the same lumbar level, between L5 and L4. Once the probe was in this position, a single image was taken.

One of the examiners was an experienced musculoskeletal sonographer, with more than 15 years’ experience, and the other was a new sonographer with 1 year of experience. The ultrasound measurements of each variable were taken three times per examiner from each side of the subject, repeating the process of probe placement for each measurement. The average value was taken as a reference because, as demonstrated by Koppenhaver et al., this optimizes the accuracy of the ultrasound measurement [23]. The measurement process by the 2 examiners was randomized to minimize possible systematic errors. It should be noted that the examiners assessed each subject on only one occasion.

Each ultrasound image obtained was assigned a reference that was recorded in a database. Subsequently, another researcher, who did not carry out the ultrasound measurements and therefore did not know to which subject each image belonged, was responsible for calculating the pennation angles and muscle thickness of the superficial and deep multifidus musculature.

All ultrasound image analyses were performed using ImageJ image analysis software [25], in this case FIJI (Fiji Is Just ImageJ) (version 2.1.0/1.53c).

**Figure 2:**
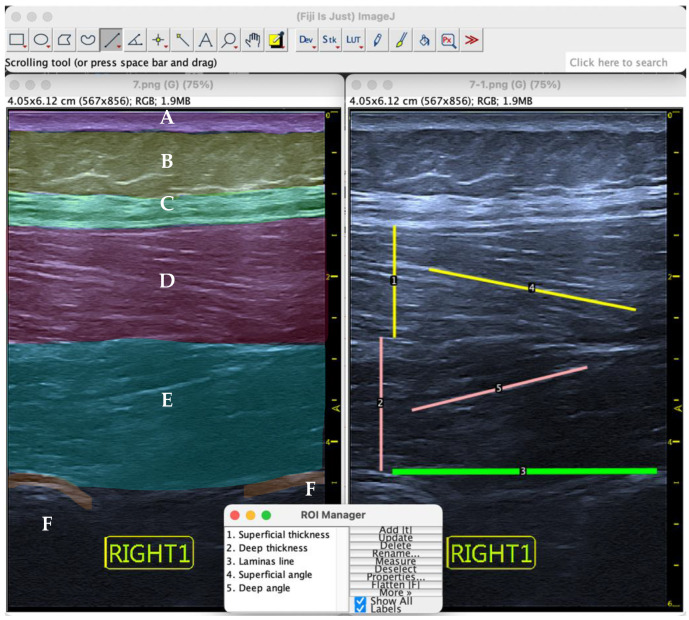
Ultrasound image of the lumbar multifidus musculature (sagittal view) during measurement of the thickness (lines 1–2) and pennation angle (lines 4–5). (**A**) Skin; (**B**) fat; (**C**) thoraco-lumbar fascia; (**D**) superficial multifidus; (**E**) deep multifidus; (**F**) laminae.

The angle of pennation was defined as the angle between the most hyperechogenic fiber of the multifidus (superficial or deep) and the baseline joining the two vertebral laminae underneath (Figure 2).

The angle of pennation was obtained through the coordinate axis of the lines using the ROI Angle Calculator tool, an Excel tool that calculates the vector of each line, the scalar product and the modulus of both and, finally, applies the cosine formula to calculate the two complementary angles that the lines form between themselves (Figure 2, lines 3 to 5). The lesser angle was always chosen to define the angle of pennation, which was always the closer of the two.

Thus, on the one hand, the pennation angle of the superficial line and the line between the laminae were compared to obtain the AP of the superficial multifidus. On the other hand, the line of the deep multifidus was compared with the same line between the laminae as a base, to obtain the pennation angle of the deep muscle. This process was carried out on the 6 images belonging to each of the subjects, taking the average value of each of the sides as the reference value.

On the other hand, for the measurement of muscle thickness, a secondary variant of the study, the same images generated for the pennation angle were used, as well as the same image analysis software.

The calculation of the thickness of both the deep and superficial multifidus was made at the L4 level. For the deep multifidus, a linear measurement was taken from the lamina of L4 to the superior border of the deep multifidus muscle—an anatomical limit differentiated by the change in the direction of the fibers (Figure 2, line 2).

Additionally, to measure the thickness of the superficial multifidus, the linear distance between the bottom of the thoracolumbar fascia and the lower edge of the superficial multifidus muscle was calculated—an anatomical limit differentiated by the change in orientation of the muscle fibers (Figure 2, line 1).

Information relevant to demographic variables such as age, height, sex, weight, Body Mass Index, or upper and lower limb dominance and regular physical activity was obtained by interviewing each patient.

Statistical analysis was performed with R Ver. 3.5.1. (R Foundation for Statistical Computing, Institute for Statistics and Mathematics, Welthandelsplatz 1, 1020 Vienna, Austria). The significance level was set at *p* < 0.05. Quantitative variables were described with mean and standard deviation and qualitative variables with absolute and relative values (%). The distribution of the quantitative variables was tested with the Kolmogorov–Smirnov test with Lillierfors correction, which showed the absence of normality. The intraclass correlation coefficient (ICC2) as relative reliability was calculated for intra- and interobserver for thickness and angle of pennation, and defined as poor (<0.5), moderate (0.5–0.75), good (0.75–0.9), or excellent (>0.9) [26], while standard error of measurement (SEM) was calculated as absolute reliability [27].

The correlation between thickness and pennation angle, and between baseline variables and thickness and pennation angle, was calculated using the Pearson or Spearman correlation matrix according to their distribution, or a polychoric matrix in the case of qualitative variables, defined as weak (<0.29), moderate (0.3–0.49), high (0.5–0.89), or very high (>0.90). Correlations between thickness and pennation angle, both superficial and deep, are displayed by scatter plots with regression lines on a graph in which the values of two variables are plotted along two axes, with the pattern of the resulting points revealing any correlation present.

## 3. Results

The sample consisted of 27 subjects aged 23.37 ± 3.35 years, with a balance of males and females, with a Body Mass Index of 22.48 ± 2.99 and practicing sport 2.81 ± 1.88 times a week (Table 1).

The results of the multifidus muscles thickness and pennation angle measurements obtained during the sampling classified by gender are detailed in Table 2.

### Reliability

All ICC were significant (*p* < 0.05), with values in the thickness of good (six cases), moderate (two cases), and poor (four cases), and in the pennation angle with good (two cases), moderate (eight cases) and poor (two cases) (Table 3).

The coefficients between thickness and pennation angle were positive, except in the correlation between left deep thickness vs. left deep pennation angle (which was negative), high (2 cases) and weak (2 cases), while high values were observed for the remaining variables (9 cases), moderate (21 cases), and weak (34 cases). Table 4 presents the high cases.

**Figure 3:**
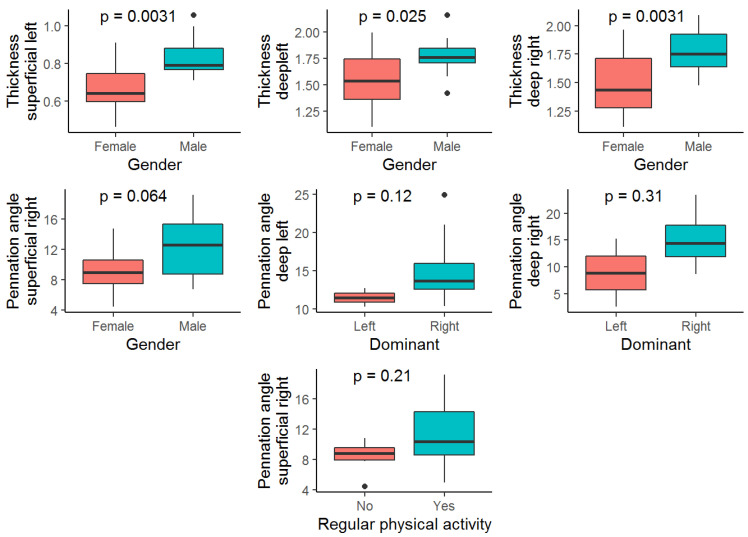
Higher correlations boxplots by gender, dominant side, and physical activity.

Secondary analyses were carried out to explore these correlations in order to identify the main correlation between gender and dominant and regular physical activity by a boxplot, as presented in Figure 3.

**Figure 4:**
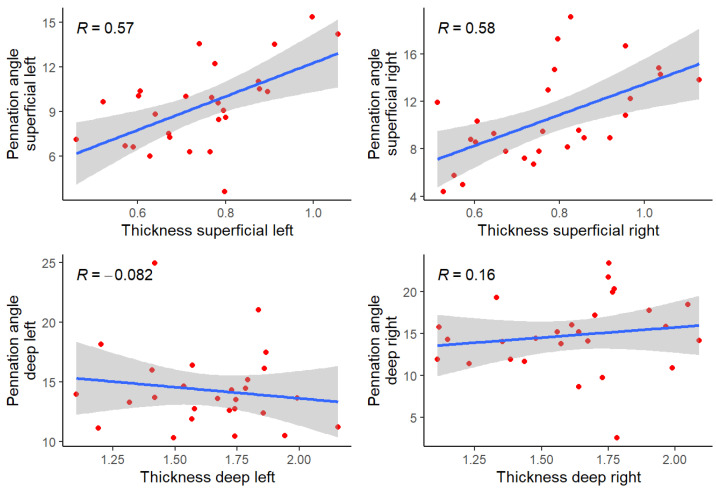
Multifidus thickness vs. pennation angle correlation. *R*: correlation coefficient.

The Pearson correlation coefficient except for pairwise that includes Pennation angle deep left variables, which shower the Spearman correlation coefficient. The scatter plots for thickness and the pennation angle are shown below (Figure 4).

## 4. Discussion

Both raters obtained excellent intra-rater reliabilities in most measurements of the pennation angle and muscle thickness variables. High correlation was obtained between superficial pennation angle and superficial thickness for left and right sides.

The multifidus muscles angle pennation is a new measurement which revealed interesting data (as detailed in Table 2) for future studies.

The disparities obtained in terms of the resulting values of intra-rater reliability, both for the angle of pennation and for the muscle thickness, could be related to the laterality of the observers, this being a conditioning factor in the process of obtaining the ultrasound.

The moderate interrater reliability obtained in most cases for the measurement of both variables studied could be linked to the fact that one of the examiners had more than 15 years of experience in ultrasound imaging, as opposed to the other observer, who lacked such experience and was a novice in performing this procedure. However, both observers obtained good intra-observer correlation indices.

However, it should be noted that when comparing the values obtained with those of other studies, in some cases the intra-rater reliability of the measurement of muscle thickness was like that obtained by Walwork et al. [28], who reported results of 2.86 ± 0.26 for experienced versus 2.82 ± 0.25 for novice raters.

No comparisons could be made regarding the angle of pennation due to the lack of studies, but we believe that a new line for research has been opened, to be developed in future studies, as the data showed a positive correlation between muscle thickness and pennation angle in the superficial multifidus muscles. However, no such correlation was observed in the deep multifidus muscles.

The positive correlation between muscle thickness and pennation angle could be explained by the fact that larger muscle size is generally associated with an increase in fascicle angle, as has been observed in soccer players and swimmers of both genders. The quadriceps vastus lateralis and gastrocnemius medialis muscles revealed increases in these variables (thickness and angle) related to strength increase [29,30,31]. This evidence demonstrated that muscle thickness relative to fascicle angles was significantly greater in the males and was muscle contraction-type dependent, finding that soccer players had greater thickness and pennation angle in medial gastrocnemius, which was associated with increased activity in comparison with untrained people [31].

On the other hand, the fact that this correlation was not reflected in the deep muscles could lead us to consider that muscle function and anatomical arrangement, which is different in deep and superficial areas [24], may influence the architecture [32]; this result would be a point for evaluation in the population with low back pain in order to determine how pain and disability can affect this correlation.

Blazevich et al. [32] also found different correlations among elements of muscle architectures between deep and superficial muscles belonging to the same muscle group. They found differences in the correlation between thickness and pennation angle that were positive between superficial quadriceps muscles, but no such correlation in the deep vastus intermedius muscle, which they attribute to the fact that the vastus intermedius muscle probably has a different function to the superficial quadriceps muscles.

Therefore, the absolute or relative architecture of a muscle cannot be used with certainty to estimate the total architecture of the muscle group. It is suggested not only that an analysis of the architecture of a muscle group that theoretically has the same function should be carried out, but that muscles should also be examined individually, because of the multiple variations that can be found.

Changes in pennation angles have been identified in pathological cases in previous research in patients with sarcopenia [18], as an associated method for early diagnosis, and multiple sclerosis patients [33] showed a decrease in this variable compared with controls. Considering these results, it is fairly probable that the pennation angle could change in people with low back pain, and our protocol and results will be useful. Kirmaci et al. [33] reported a decrease in this variable compared with controls.

Based on this reasoning, we can assume that the difference between the correlations of the architecture of deep and superficial multifid muscles is probably because they have different functions. However, the assumption that architecture reflects function has not yet been empirically tested, and this should be proposed for future studies based on the data obtained.

Overall, a gender difference was found in the muscle thickness of both deep and superficial muscles. The fact that muscles are larger in men than in women is a consistent finding in the literature [29,34,35,36]. Stokes et al. [34], in their study, relate a larger muscle size in men to differences in body mass, and this must be taken into account when discussing pathological cases. However, it is interesting to note that, in our study, the angle of pennation did not show a significant difference between the two genders.

Boxplot analyses demonstrated a higher association in women for deep and superficial multifidus on the left side and deep multifidus on the right side. However, the pennation angle on the superficial left side showed a higher association for men. Physical activity resulted in a higher association for pennation angle of superficial multifidus. These results will inform a hypothesis for future studies with a larger sample size and will be compared with pathological cases to determine the implication for clinical settings.

Regarding the limitations of this study, it should be noted that the measurements were taken on a single day instead of on alternate days separated in time, and the raters were both expert and novice. It would be interesting in the future to carry out the study with a larger sample of subjects and protocolized sampling on alternate days. Additionally, correlation with a gold standard method (MRI or CT) would be interesting for pennation angle, since thickness has been demonstrated to have an influence.

This research provides the protocol necessary for future studies, where it could be applied to monitor the effects of exercise-based therapy, neuromodulation, manual therapy or others; the impact of pain in this region; and its correlation with disability or its relationship with other pathologies.

## 5. Conclusions

The ultrasound measurement protocol designed for the measurement of the pennation angle of the lumbar multifidus musculature showed excellent intra-rater reliability for both superficial and deep lumbar multifidus muscles, as well as moderate interrater reliability. In this paper, a new variable for architectural morphology was described for future studies, and normative ranges were reported.

In addition, we observed a high correlation of pennation angle with the variables muscle thickness, gender, laterality, and regular physical activity; and of muscle thickness with the variables pennation angle, gender, and weight.

## Figures and Tables

**Table 1:** Baseline characteristics of participants.

*n*		27
Gender, *n* (%)	Female	15 (55.6)
	Male	12 (44.4)
Age		23.37 ± 3.35
Height (cm)		173.81 ± 11.00
Weight (kg)		68.13 ± 12.25
Body Mass Index		22.48 ± 2.99
Dominant, *n* (%)	Left	2 (7.4)
	Right	25 (92.6)
Regular physical activity yes/no, *n* (%)	No	6 (22.2)
	Yes	21 (77.8)
Times per week		2.81 ± 1.88

Data expressed with mean ± standard deviation or with absolute and relative values (%).

**Table 2:** Ultrasound morphology by gender.

	Observer 1		Observer 2	
	Female	Male	Female	Male
* n *	15	12	15	12
Thickness superficial left	0.63 ± 0.11	0.79 ± 0.14	0.71 ± 0.17	0.87 ± 0.17
Thickness superficial right	0.67 ± 0.16	0.76 ± 0.12	0.77 ± 0.23	0.92 ± 0.20
Thickness deep left	1.44 ± 0.29	1.59 ± 0.23	1.63 ± 0.32	1.94 ± 0.22
Thickness deep right	1.36 ± 0.29	1.75 ± 0.28	1.61 ± 0.31	1.81 ± 0.22
Pennation angle superficial left	7.31 ± 3.26	9.78 ± 3.48	9.51 ± 3.16	11.29 ± 3.34
Pennation angle superficial right	8.14 ± 2.63	11.33 ± 4.64	10.12 ± 3.91	13.38 ± 5.17
Pennation angle deep left	13.47 ± 3.56	13.42 ± 4.00	15.71 ± 4.89	14.43 ± 2.82
Pennation angle deep right	12.42 ± 4.12	15.13 ± 5.05	15.17 ± 4.92	17.02 ± 5.60

Data expressed with mean ± standard deviation.

**Table 3:** Intraclass correlation coefficient results.

		Thickness						Pennation Angle			
				ICC (95% CI)	ICC Categorical	^a^*p* Value	SEM (95% CI)	ICC (95% CI)	ICC Categorical	^a^*p* Value	SEM (95% CI)
Superficial	Left	Intraobserver	Observer 1	0.851 (0.74, 0.923)	Good	<0.001	0.059 (0.047, 0.071)	0.801 (0.665, 0.896)	Good	<0.001	1.714 (1.354, 2.074)
	Left		Observer 2	0.842 (0.728, 0.918)	Good	<0.001	0.076 (0.059, 0.094)	0.669 (0.478, 0.817)	Moderate	<0.001	2.187 (1.707, 2.667)
	Right		Observer 1	0.776 (0.627, 0.881)	Good	<0.001	0.076 (0.059, 0.093)	0.667 (0.476, 0.816)	Moderate	<0.001	2.591 (1.916, 3.266)
	Right		Observer 2	0.711 (0.535, 0.843)	Moderate	<0.001	0.138 (0.096, 0.181)	0.838 (0.721, 0.916)	Good	<0.001	1.979 (1.574, 2.385)
	Left	Interobserver		0.392 (0.046, 0.661)	Poor	0.012	0.127 (0.088, 0.166)	0.304 (−0.038, 0.595)	Poor	0.037	2.767 (1.962, 3.571)
	Right			0.458 (0.052, 0.724)	Poor	0.001	0.128 (0.087, 0.169)	0.514 (0.171, 0.745)	Moderate	0.001	2.877 (2.044, 3.711)
Deep	Left	Intraobserver	Observer 1	0.764 (0.61, 0.874)	Good	<0.001	0.144 (0.113, 0.174)	0.556 (0.337, 0.744)	Moderate	<0.001	2.943 (2.175, 3.711)
	Left		Observer 2	0.886 (0.798, 0.942)	Good	<0.001	0.112 (0.085, 0.14)	0.702 (0.522, 0.837)	Moderate	<0.001	2.474 (1.842, 3.106)
	Right		Observer 1	0.794 (0.653, 0.891)	Good	<0.001	0.171 (0.131, 0.211)	0.709 (0.532, 0.842)	Moderate	<0.001	2.817 (2.172, 3.462)
	Right		Observer 2	0.708 (0.531, 0.841)	Moderate	<0.001	0.172 (0.124, 0.22)	0.738 (0.57, 0.86)	Moderate	<0.001	2.836 (2.101, 3.572)
	Left	Interobserver		0.389 (−0.041, 0.687)	Poor	0.002	0.202 (0.143, 0.262)	0.4 (0.055, 0.667)	Poor	0.011	2.936 (2.166, 3.706)
	Right			0.494 (0.137, 0.735)	Poor	0.001	0.212 (0.153, 0.271)	0.519 (0.171, 0.749)	Moderate	0.001	3.246 (2.329, 4.163)

ICC (95% CI): intraclass correlation coefficient (95% confidence interval); SEM (95% CI): standard error of measurement (95% confidence interval) expressed in centimeters. ^a^ Significant if *p* < 0.05.

**Table 4:** Pairwise correlation coefficient results.

Thickness vs. Pennation Angle		
Thickness superficial left vs. Pennation angle superficial left	0.573	High
Thickness superficial right vs. Pennation angle superficial right	0.576	High
**Pennation angle and thickness vs. Baseline variables**		
Thickness superficial left vs. Gender	0.730	High
Thickness deep left vs. Gender	0.594	High
Thickness deep right vs. Gender	0.677	High
Pennation angle superficial right vs. Gender	0.530	High
Thickness deep left vs. Weight (kg)	0.684	High
Thickness deep right vs. Weight (kg)	0.621	High
Pennation angle deep left vs. Dominant	0.708	High
Pennation angle deep right vs. Dominant	0.576	High
Pennation angle superficial right vs. Regular physical activity (yes/no)	0.517	High
